# Age-dependent inflammatory response is altered in an ex vivo model of bacterial pneumonia

**DOI:** 10.1186/s12931-023-02609-w

**Published:** 2024-01-04

**Authors:** Charline Sommer, Stella Marie Reamon-Buettner, Monika Niehof, Christina Beatrix Hildebrand, Armin Braun, Katherina Sewald, Susann Dehmel, Christina Brandenberger

**Affiliations:** 1 Fraunhofer Institute for Toxicology and Experimental Medicine, Hannover, Germany; 2 Member of Fraunhofer International Consortium for Anti-Infective Research (iCAIR), Member of Fraunhofer CIMD, Hannover, Germany; 3 Member of the German Center for Lung Research (DZL), Biomedical Research in Endstage and Obstructive Lung Disease Hannover (BREATH) research network, Hannover, Germany; 4 Institute for Functional and Applied Anatomy, Hannover Medical School, Hannover, Germany; 5 Institute of Functional Anatomy, Charité – Universitätsmedizin Berlin, Philippstr. 11, Berlin, 10117 Germany

**Keywords:** Inflamm-aging, Neutrophils, *Pseudomonas aeruginosa*, Precision-cut lung slices

## Abstract

**Background:**

Aging is associated with an increased incidence and mortality of *Pseudomonas aeruginosa*-induced pneumonias. This might be partly due to age-dependent increases in inflammatory mediators, referred to as inflamm-aging and a decline in immune functions, known as immunosenescence. Still, the impact of dysregulated immune responses on lung infection during aging is poorly understood. Here, we aimed to mimic inflamm-aging using ex vivo precision-cut lung slices (PCLS) and neutrophils – as important effector cells of innate immunity – from young and old mice and investigated the influence of aging on inflammation upon infection with *P. aeruginosa* bacteria.

**Methods:**

Murine PCLS were infected with the *P. aeruginosa* standard lab strain PAO1 and a clinical *P. aeruginosa* isolate D61. After infection, whole-transcriptome analysis of the tissue as well as cytokine expression in supernatants and tissue lysates were performed. Responses of isolated neutrophils towards the bacteria were investigated by quantifying neutrophil extracellular trap (NET) formation, cytokine secretion, and analyzing expression of surface activation markers using flow cytometry.

**Results:**

Inflamm-aging was observed by transcriptome analysis, showing an enrichment of biological processes related to inflammation, innate immune response, and chemotaxis in uninfected PCLS of old compared with young mice. Upon *P. aeruginosa* infection, the age-dependent pro-inflammatory response was even further promoted as shown by increased production of cytokines and chemokines such as IL-1β, IL-6, CXCL1, TNF-α, and IL-17A. In neutrophil cultures, aging did not influence NET formation or cytokine secretion during *P. aeruginosa* infection. However, expression of receptors associated with inflammatory responses such as complement, adhesion, phagocytosis, and degranulation was lower in neutrophils stimulated with bacteria from old mice as compared to young animals.

**Conclusions:**

By using PCLS and neutrophils from young and old mice as immunocompetent ex vivo test systems, we could mimic dysregulated immune responses upon aging on levels of gene expression, cytokine production, and receptor expression. The results furthermore reflect the exacerbation of inflammation upon *P. aeruginosa* lung infection as a result of inflamm-aging in old age.

**Supplementary Information:**

The online version contains supplementary material available at 10.1186/s12931-023-02609-w.

## Introduction

In the elderly, respiratory infections are the leading infection-related cause of death worldwide with increasing incidences of influenza, COVID-19, and bacterial pneumonias [1–3]. In bacterial lung infections, the gram-negative *Pseudomonas aeruginosa* is, after *Staphylococcus aureus*, the second major cause of ventilator-associated and hospital-acquired pneumonia [4, 5] and particularly elderly individuals show a growing risk for developing *P. aeruginosa* lung infections [6].

With advancing age, the immune function declines, a feature known as ‘immunosenescence’ [7], that is accompanied by a dysregulated chronic low-grade inflammation referred to as ‘inflamm-aging’ [8]. Although inflamm-aging is a heterogenic phenomenon with tissue-specific attributes, some pro-inflammatory cytokines including tumor necrosis factor-α (TNFα), interleukin-6 (IL-6), and IL-8 are classically associated with inflamm-aging and increasingly observed in serum and lungs of the elderly [9, 10]. This age-related increase was shown to be associated with an increased risk of developing community-acquired pneumonia in a longitudinal study over 6.5 years [11]. Furthermore, the pro-inflammatory milieu correlates with an increased neutrophil influx observed in lungs of many healthy, clinically normal elderly individuals [9, 10, 12]. Hence, these changes in immunity could contribute to the increased morbidity and mortality rates of *P. aeruginosa* pneumonias in the elderly [6, 13, 14].

One hallmark of the inflammatory response towards pulmonary *P. aeruginosa* infections is the early, massive influx of neutrophils into the lungs [6, 15]. Their primary function is to eliminate the pathogens with phagocytosis, secretion of bacterio-toxic granules, or by secreting neutrophil extracellular traps (NETs) [16, 17], which trap and eliminate the bacteria [16–19]. However, although neutrophils possess an important function in pathogen elimination, their defense mechanism not only damages invading bacteria, but also the lung tissue itself, contributing to the severity of pneumonia due to lung injury [20, 21]. Hence, their presence in lung infection represents a double-edged sword. With aging, neutrophil effector function often is reported to be decreased, including migration towards inflammatory stimuli [22, 23], phagocytosis [24, 25], and intracellular killing [23]. However, although neutrophils are key cells in *P. aeruginosa* lung infections, little is known about the impact of age on neutrophil function in response to the bacterium.

The growing incidence as well as the high mortality of *P. aeruginosa*-induced pneumonias makes it an especially challenging disease in the elderly. Still, although several studies report inflamm-aging in healthy elderly, changes in the host immune response towards *P. aeruginosa* pneumonias upon aging are not well characterized. Understanding immunological changes within the lungs as well as neutrophil response upon aging could be key in improving treatment strategies and reducing mortality rates in elderly individuals suffering from *P. aeruginosa* lung infections. Therefore, we aimed to mimic inflamm-aging and its impact on *P. aeruginosa* infection by using ex vivo precision-cut lung slices (PCLS) and neutrophils of young and old mice. Within PCLS, all lung-resident cells are present, enabling reflection of in vivo immunological changes and the investigation of complex immune responses as demonstrated previously [26]. Using ex vivo lung slices and isolated cells as a suitable testing system to study mechanisms of lung infection in aging, we hypothesized that inflamm-aging is promoted upon *P. aeruginosa* infection of PCLS and neutrophils. Results gained from this study shall provide new insights into immunological changes during *P. aeruginosa* lung infections with aging and, with that, build a basis for further research to improve course and outcome of the disease in the elderly.

## Methods

### Animals

Young (10 to 15 weeks) and old (18 to 21 months) male C57BL/6NCrl-mice were sacrificed for organ removal in accordance with the German Animal Protection Law and European Council Directive on the protection of animals used for scientific purposes (2010/63/EU). A limitation of our study is the usage of male mice only without including female animals.

### Bacterial strains

A laboratory reference strain PAO1 (DSZM #19,880, DSMZ Braunschweig, Germany) and a clinically isolated *P. aeruginosa* strain, D61, from a cystic fibrosis patient (kindly provided by Tümmler and colleagues, Hannover Medical School, Germany) [27] were used and cultured as described in the online supplement.

### Preparation and infection of precision-cut lung slices

Murine PCLS were prepared as described previously [28, 29], with some modifications (see online supplement). Two PCLS/well were infected in DMEM/F-12 with 1 × 10^5^ colony forming units (CFU) *P. aeruginosa* PAO1 or D61 or left uninfected (control) and incubated at 37 °C, 5% CO_2_ (technical triplicates). For better comparability, PCLS of young and old mice were always processed and infected simultaneously in experimental replicates. Eight hours post infection (p.i.), supernatants and tissue lysates of technical replicates were pooled, supplemented with protease inhibitor cocktail (Sigma-Aldrich), and aliquots were stored at -80 °C until usage.

### RNA isolation and transcriptomics

RNA was isolated according to an optimized protocol for PCLS [30]. Transcriptome analyses were done using the Affymetrix GeneChip™ Whole Transcript (WT) PLUS Reagent Kit and the GeneChip™ mouse Clariom™ S Arrays according to the manufacturer’s recommendation (ThermoFisher Scientific) (see online supplement). Database for Annotation, Visualization and Integrated Discovery (DAVID) online tool [31, 32] and Enrichr [33–35] were used for enrichment analysis (gene ontology term) of biological processes.

### Cytokine measurements

Cytokines were measured in PCLS supernatants and lysates using DuoSet ELISA Kits (IL-6, CXCL1, CCL3; biotechne) and Meso Scale Discovery (MSD) assay (TNF-α, IL-1β, CCL20, IL-17A; Meso Scale Diagnostics) according to the manufacturer’s instructions. The sum of extrinsic und intrinsic cytokine concentrations was normalized to the total protein content determined by bicinchoninic acid (BCA) assay (Pierce^Tm^ BCA Protein Assay Kit). Cytokines in the supernatant of stimulated neutrophils were measured with the LEGENDplex cytokine array (13-plex virus response panel, BioLegend) according to the supplier’s protocol.

### Isolation of neutrophils

Neutrophils were isolated from the bone marrow of young and old mice as described previously [36]. Isolated cells were counted, tested for viability with trypan blue and purity by generating cytospins stained with the DiffQuick staining kit (Medion Diagnostics). Neutrophils from young and old mice were always processed simultaneously within each experimental replicate.

### Quantification of NETs

NET formation of 100,000 neutrophils/well was quantified by measuring fluorescence of SYTOX green, staining extracellular DNA, for 4 h using the Tecan reader (excitation: 485 nm, emission: 535 nm) (for details see online supplement).

### Visualization of NETs

Scanning electron microscopy (SEM) imaging of NETs was performed as previously described [37] after co-culturing neutrophils and bacteria at a multiplicity of infection (MOI) of 10 for 4 h (see online supplement). Imaging was done at 10 kV and 1270 x and 5000 x with the Zeiss Crossbeam 540 (Carl Zeiss Microscopy GmbH).

### Flow cytometry of neutrophil activation

For flow cytometric analysis, 100,000 neutrophils/sample were incubated with bacteria at a MOI of 5. After 4 h, supernatant of technical duplicates was pooled and supplemented with protease inhibitor cocktail for cytokine secretion analysis and cells were stained for surface expression markers CD11b, CD32, CD88, CD16, and CD32 (details in online supplement).

### Co-culture of PCLS and neutrophils

PCLS and neutrophils from young and old donor mice were co-cultured in a 2 × 2 design. Two PCLS were combined with 100,000 neutrophils per well and infected with 1 × 10^5^ CFU PAO1 in technical duplicates, as described. Notably though, old mice were significantly older than in previous experiments (26 to 27 months old), due to availability issues. Co-culture was performed for 4 h before supernatants were harvested, and tissue was lysed as described. Total cytokine content was quantified using the LEGENDplex cytokine array (13-plex virus response panel, BioLegend) according to the supplier’s protocol and the sum of extrinsic und intrinsic cytokine concentrations was normalized to the total protein content as described.

### Statistics

Statistical analyses were done with the SigmaPlot® software, version 13.0.0.83 (SYSTAT® Software Inc.). Data was analyzed by two-way ANOVA followed by a multiple-pairwise comparison Bonferroni-test. If the normality test failed, data were transformed with the natural logarithm (ln) prior to analysis. Alternatively, Mann-Whitney Rank Sum Test was applied with Bonferroni correction. Differences were considered as significant for *p* < 0.05. Graphs depicted in boxplots were created using Prism 9 (GraphPad). Box plots display the median with the 25th and 75th percentile and whiskers mark the lowest and highest value. For co-culture experiments, cytokine levels were analyzed by 3-way ANOVA to test for age-effects in PCLS, age-effects in neutrophils, and PAO1-infection effects (*p* < 0.05).

## Results

### Immune-related processes are age-dependently regulated in PCLS, both under uninfected conditions and upon *P. aeruginosa* infection

To investigate the impact of the aging host immune response towards bacterial infections, lung slices of young and old mice were infected ex vivo with *P. aeruginosa* and comparatively analyzed. The infection was performed with PAO1 as a standard laboratory strain as well as D61, isolated from a chronically infected cystic fibrosis patient. Genome-wide transcriptome analysis was performed after ex vivo infection of PCLS from young versus old mice. Notably, a difference was observed when comparing uninfected control conditions with an unbiased clustering approach, as samples of young vs. old mice clustered into two distinct groups (Supplementary Fig. S1). This supports the notion that aging already alters baseline conditions in the lung tissue.

**Fig. 1:**
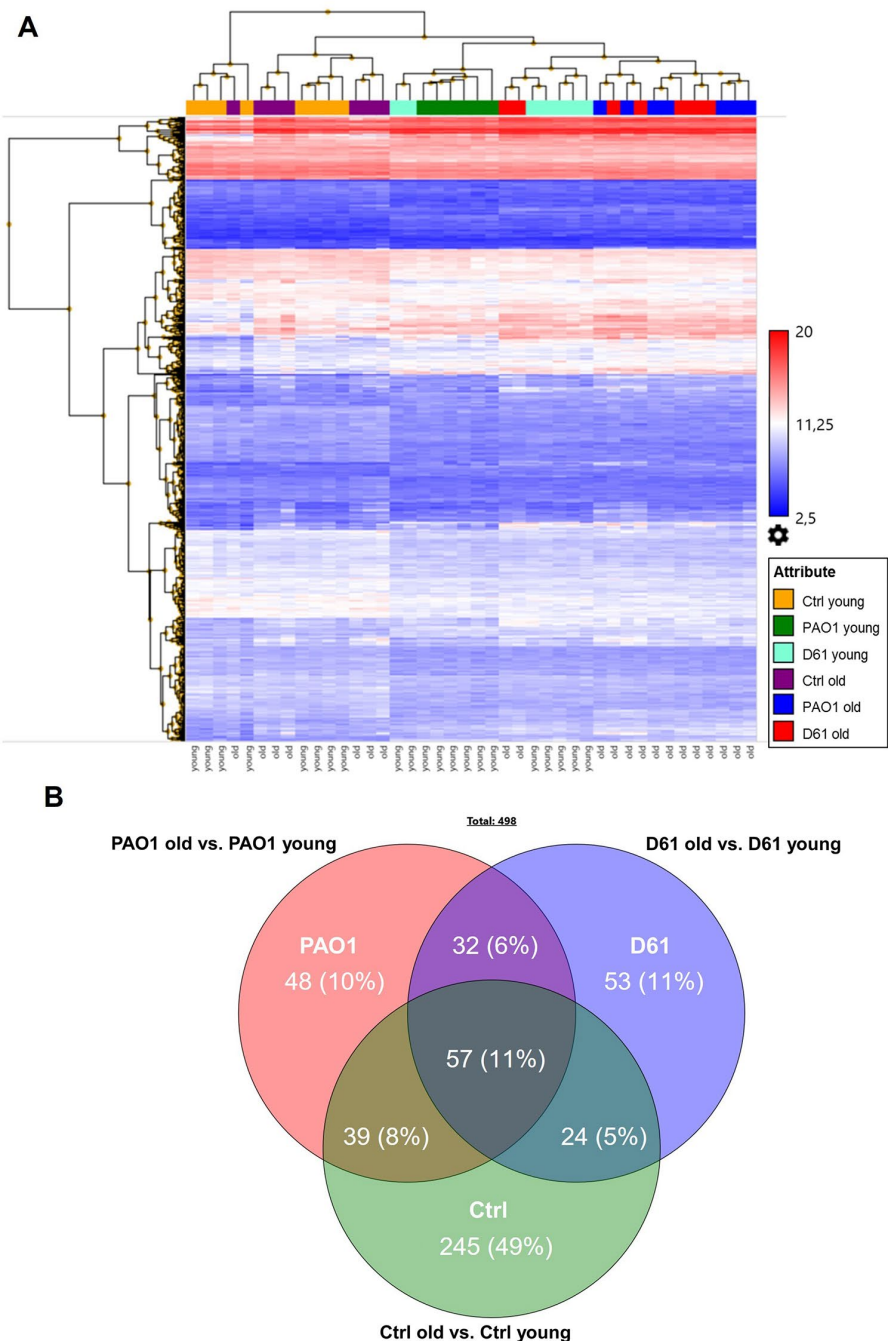
**Age-dependent gene expression in PCLS under control conditions and upon** ***P. aeruginosa*** **infection**. PCLS of young and old mice were infected with 1 × 10^5^ CFU/well PAO1 or D61 or cultured without bacteria (Ctrl), washed 1 h p.i. and transferred into new wells. Infection was continued for 7 h and genome-wide transcriptome analysis was performed on tissue slices of old vs. young mice. (**A**) Overview of gene expression analysis depicted as unsupervised hierarchical clustering. (**B**) Venn diagram showing the distribution of differentially expressed genes (≥ 2-fold change, *p* < 0.05) of lung tissue of old compared to young mice

Upon *P. aeruginosa* infection with PAO1 or D61, separate clusters of uninfected and infected control groups were present in the unsupervised clustering (Fig. 1A). Strikingly, the infected cluster itself split into distinct clusters between young and old, indicating age-dependent regulation of genes upon infection with either of the two *P. aeruginosa* strains. When comparing gene signatures from lung tissue of old versus young mice in each of the three conditions (uninfected control, PAO1- or D61-infected), a total of 498 genes were found to be differentially regulated (> 2-fold change, *p* < 0.05; Fig. 1B). Most differentially expressed genes (DEGs) (49%) were specifically regulated in lung tissue from old vs. young mice without bacteria, confirming the strong age-effect under baseline conditions. On the other hand, 10% (PAO1) and 11% (D61) DEGs were unique for the respective bacteria, indicating additional strain-specific differences within age-regulated genes.

**Fig. 2:**
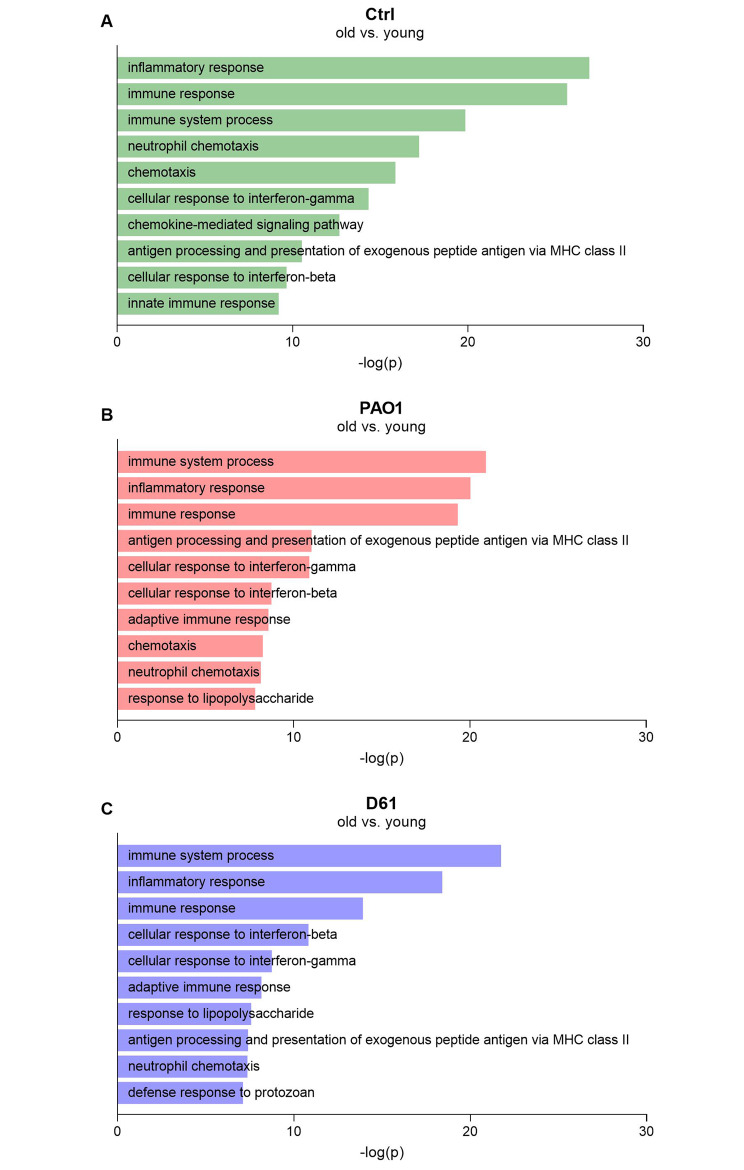
**Biological processes specifically affected by age include immune-related processes in uninfected and** ***P. aeruginosa*-infected PCLS**. PCLS of young and old mice were infected with 1 × 10^5^ CFU/well PAO1 or D61 or cultured without bacteria (Ctrl). After 8 h, whole genome analysis was performed. Differentially regulated genes (≥ 2-fold change, *p* < 0.05) were analyzed in DAVID for enrichment analysis of biological process ontology. The top 10 biological processes are displayed for Ctrl (**A**), PAO1-infected (**B**), and D61-infected PCLS (**C**), ranked based on their *p*-value

To determine biological processes affected by age, we performed gene ontology-enrichment analysis of the DEGs (≥ 2-fold change, *p* < 0.05) in DAVID. Of note, for the control conditions, only immune-related processes such as ‘immune response’, ‘immune system process’, and ‘chemokine-mediated signaling pathway’ were among the top 10 biological processes, with age-related ‘inflammatory response’ at the top of the list (Fig. 2A). This finding strongly indicates inflamm-aging within the ex vivo lung tissue. Likewise, immune-related processes were among the top 10 list for PAO1- and D61-infected PCLS of old compared to young mice. As expected, certain immune processes towards bacteria were also affected, such as ‘response to lipopolysaccharide’ (Fig. 2B, C). Similarly, when using Enrichr as an alternative gene set enrichment analysis software, gene ontology biological processes related to immunity were significantly affected during aging, further supporting inflamm-aging under control conditions and upon *P. aeruginosa* infection (Supplementary Fig. S2).

DAVID analysis further indicated changes in biological processes related to adaptive immunity, including ‘adaptive immune response’ and ‘antigen processing and presentation of exogenous peptide antigen via MHC class II’. This is in line with the observation that immunoglobulin-associated genes were among the top-regulated DEGs of lung slices from old vs. young mice, including *Jchain* or Fc receptors for IgE and IgG (*Fcgr2b*, *Fcer1g, Fcgr3*), both under uninfected conditions and upon *P. aeruginosa* infection (Supplementary Tables S1, S2, S3).

**Fig. 3:**
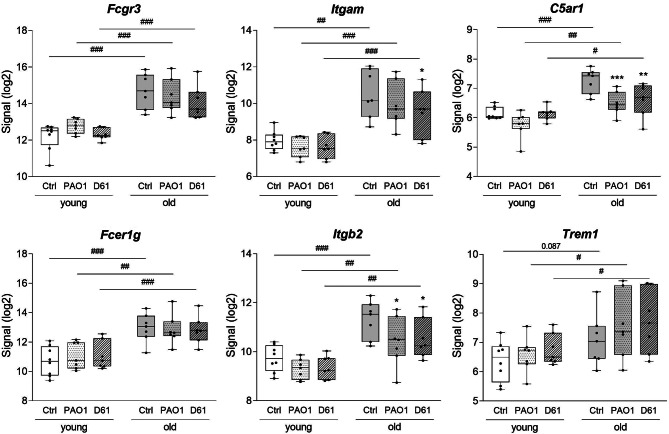
**Expression of receptors associated with immune cell chemotaxis are age-dependently regulated**. PCLS of young and old mice were infected with 1 × 10^5^ CFU/well PAO1 or D61 or cultured without bacteria (Ctrl). Whole genome analysis was performed 8 h p.i. Differentially regulated genes (≥ 2-fold change, *p* < 0.05) were analyzed in DAVID for enrichment analysis of biological process ontology and signal intensity of chosen genes encoding for receptors associated with ‘neutrophil chemotaxis’ are shown. * *p* < 0.05, ** *p* < 0.01, *** *p* < 0.001 compared with respective control (Ctrl) within one age group. # *p* < 0.05, ## *p* < 0.01, ### *p* < 0.001, between the two age groups, based on the *p*-value analyzed using the Transcriptome Analysis Console Software (TAC 4.0, Thermo Fisher Scientific). All other conditions are not significant

Notably, in infected and uninfected PCLS, chemotaxis and neutrophil chemotaxis were among the top 10 biological processes affected by age. Within these processes, expression of genes encoding for receptors associated with neutrophil and macrophage chemotaxis was increased in an age-dependent manner (Fig. 3). These receptors are associated with general activation of innate immune cells (*Fcer1g*), phagocytosis (*Fcgr3*: CD16, *Itgam*: CD11b), formation of complement receptor complexes (*C5ar1*: CD88, *Itgb2*: CD18) or amplifying production of pro-inflammatory cytokines induced by toll-like receptors (*Trem1*).

Additionally, in line with a strong age-effect on ‘inflammatory response’ within DAVID, an age-dependent gene expression of pro-inflammatory cytokines was found (Table 1). Under control conditions, cytokines typically associated with inflamm-aging, namely *Il1b*, *Il6*, and *Cxcl1* were significantly up-regulated in lung tissue from old compared to young mice. Upon infection, the expression of *Il17a* increased in an age-dependent manner, leading to significantly elevated levels in *P. aeruginosa*-infected PCLS of old versus young mice.

**Table 1:** **Expression signal (log2) and fold change of selected pro-inflammatory cytokines**. * marks significant differences of ‘old’ compared to ‘young’ in the same condition (*p* < 0.05), based on the *p*-value analyzed using the Transcriptome Analysis Console Software (TAC 4.0, Thermo Fisher Scientific)

Gene	Ctrl young	Ctrl old	fold change	*p*-value	PAO1 young	PAO1 old	fold change	*p*-value	D61 young	D61 old	fold change	*p*-value
*Tnf*	8.71	9.99	2.4	0.0512	12.01	13.84	3.6	0.0342*	13.96	15.13	2.3	0.1439
*Il1b*	13.39	15.19	3.5	0.0181*	15.84	17.47	3.1	0.0430*	16.66	18.10	2.7	0.1129
*Il6*	13.66	14.72	2.1	0.0391*	15.92	16.38	1.4	0.1036	16.96	17.65	1.6	0.2281
*Ccl3*	14.41	15.54	2.2	0.0922	17.27	18.48	2.3	0.2546	18.56	19.23	1.6	0.5090
*Ccl20*	9.00	6.61	-5.3	0.8400	12.44	14.05	3.0	0.6498	13.70	13.78	1.1	0.7384
*Cxcl1*	15.23	16.27	2.1	0.0046*	18.23	18.60	1.3	0.4739	18.55	18.43	-1.1	0.8377
*Il17a*	6.30	6.22	-1.1	0.7365	6.54	7.88	2.5	0.0127*	6.89	8.23	2.5	< 0.001*

**Fig. 4:**
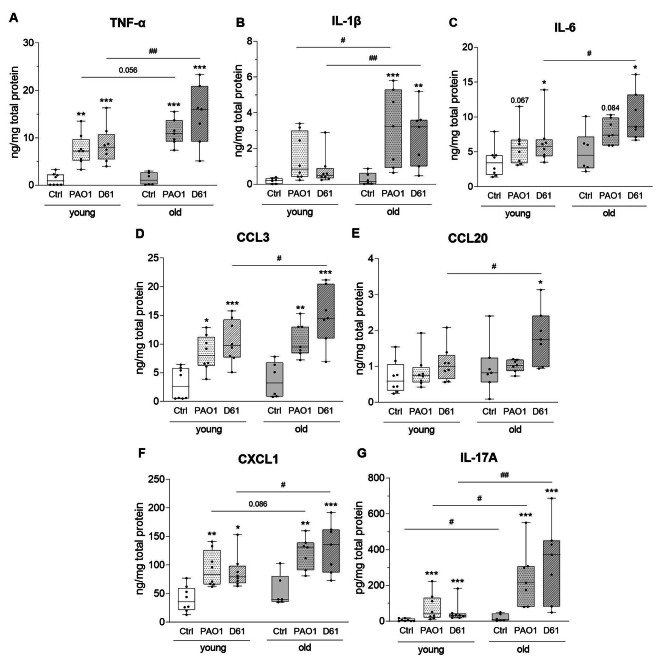
**Pro-inflammatory cytokines of PCLS are increased in an age-dependent manner upon infection with** ***P. aeruginosa***. PCLS were infected with *P. aeruginosa* PAO1 or D61 for 8 h. Supernatants and lysates of technical duplicates (Ctrl) or triplicates (PAO1 and D61) were pooled and cytokine levels were measured. The sum of extrinsic and intrinsic protein levels of TNF-α (**A**), IL-1β (**B**), IL-6 (**C**), CCL3 (**D**), CCL20 (**E**), CXCL1 (**F**), and IL-17A (**G**) is depicted in relation to the total amount of protein. * *p* < 0.05, ** *p* < 0.01, *** *p* < 0.001, compared with respective control (Ctrl) within one age group. # *p* < 0.05, ## *p* < 0.01, between the two age groups. All other conditions are not significant

The production of pro-inflammatory cytokines and chemokines was further analyzed on protein level. Control conditions without bacteria revealed no age-dependent differences in total protein content of TNF-α, IL-1β, IL-6, CCL3, CCL20, or CXCL1 (Fig. 4A-F). Amount of IL-17A, however, was significantly increased when investigating PCLS of old compared to young mice (Fig. 4G). PCLS reflect a low tissue volume and, thus, a low number of immune cells is present (Supplementary Fig. S3). Therefore, the relatively short cultivation period might not be sufficient to induce age-dependent differences of most cytokines on protein level without stimulation of the tissue.

Upon *P. aeruginosa* infection of lung tissue from old mice, production of all pro-inflammatory mediators was increased compared to PCLS of young mice infected with the same bacterial strain. Notably, these differences were mostly significant for the clinical isolate D61 but not for the laboratory strain PAO1. Increased cytokine production was not due to differences in cell amount as shown by comparable protein content of PAO1- and D61-infected PCLS (Supplementary Fig. S4) and did not affect viability of tissue slices. However, bacterial load was lower in D61- compared to PAO1-infected PCLS, which seemed to be due to a lower growth of the clinical isolate (Supplementary Fig. S5), indicating not only age- but also strain-specific differences in host immune responses towards the host-adapted strain. In contrast, fold changes of cytokine gene expression of old compared to young were more pronounced in PAO1- than D61-infected PCLS with significant increases in *Tnf* and *Il1b* expression (Table 1). Together, these data show increased inflammatory responses in lung tissue upon aging which is further increased upon *P. aeruginosa* infection on transcriptome and protein level. A summary of age-dependently regulated genes and proteins can be found in Supplementary Table S4.

### Neutrophil surface activation markers are age-dependently regulated upon *P. aeruginosa infection*, while NET formation is not

Neutrophils have a crucial role in microbial defense, however, they also contribute to the development of injury and tissue damage in infection [20, 21]. In PCLS, where neutrophils are scarcely present, an increased expression of pro-inflammatory cytokines and chemokines related to neutrophil recruitment and activation such as CXCL1, CCL3, or IL-17A was measured, particularly in PCLS of old mice upon infection with D61. Additionally, we found gene expression of receptors related to neutrophil chemotaxis to be differentially regulated in PCLS of old vs. young mice. We therefore further analyzed age-dependent neutrophil activation and stimulation ex vivo with a focus on cytokine secretion, NET formation, and surface molecule expression to investigate their potential contribution to pulmonary defense and inflammation with age.

**Fig. 5:**
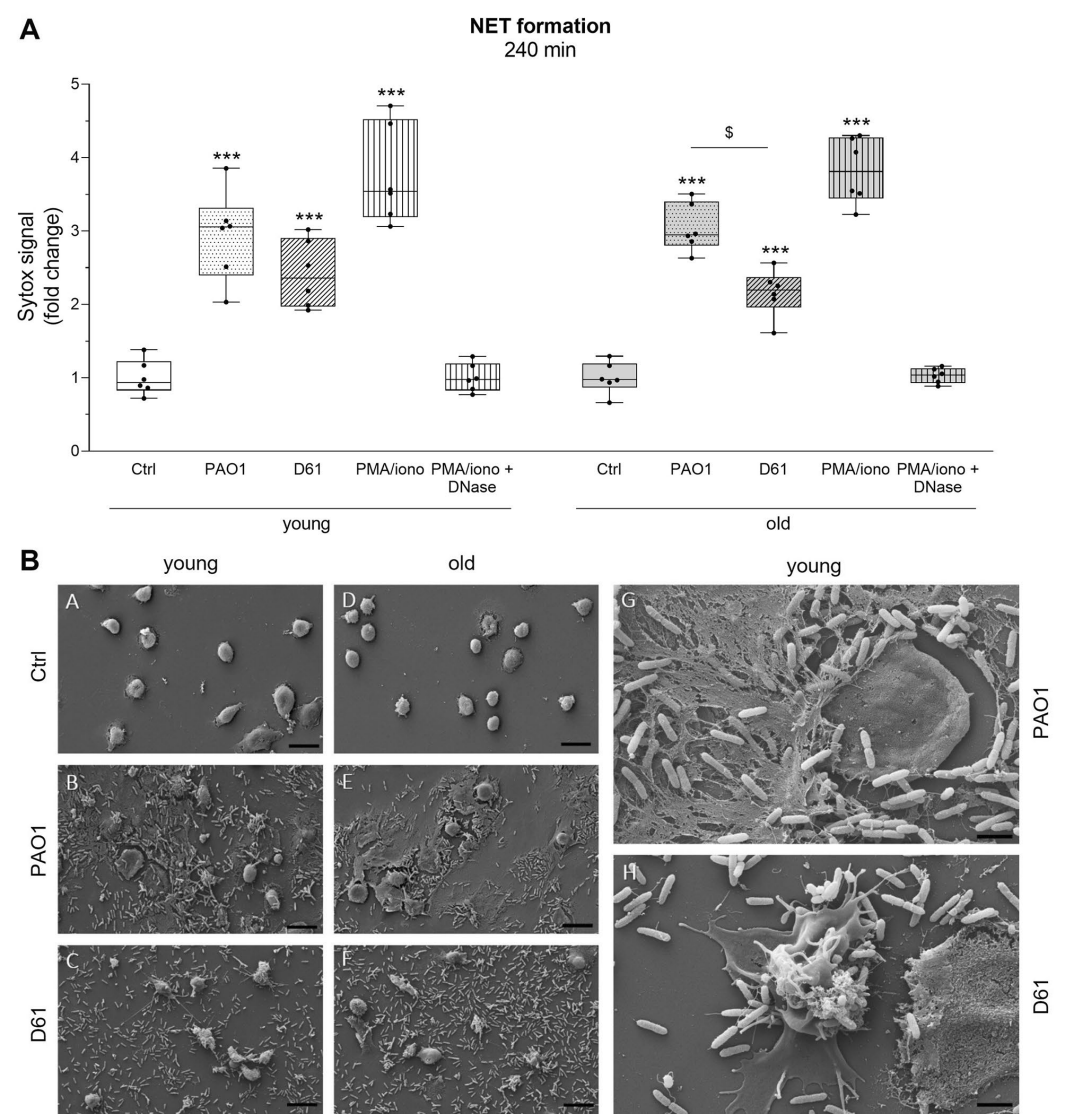
**NET formation is lower in response to the clinical** ***P. aeruginosa*** **isolate and differs morphologically**. Neutrophils of young and old mice were left unstimulated (Ctrl), stimulated with a PMA/ionomycin cocktail, PMA/ionomycin plus DNase I or bacteria (PAO1 or D61) at MOI of 5 for 4 h. (**A**) NET formation was monitored by adding SYTOX green and measurement of relative fluorescence units (RFU). SYTOX green signal was normalized to unstimulated control neutrophils (Ctrl) to yield the fold change compared with Ctrl. n = 6 mice per age group. *** *p* < 0.001, compared with respective control within one age group. $ *p* < 0.05, between two bacterial strains within one age group. All other conditions not significant. No differences between the two age groups. (**B**) NET formation was investigated using scanning electron microscopy. Representative images are shown for neutrophils of young (**A**-**C**) and old mice (**D**-**F**), scale bar = 10 μm. NET formation of neutrophils from young mice in response to PAO1 (**G**) or D61 (**H**) is shown as a close-up image, scale bar = 2 μm

NET formation was induced upon co-culture of *P. aeruginosa* bacteria with neutrophils from young and old mice as visualized by fluorescence microscopy showing DNA protrusions speckled with myeloperoxidase (Supplementary Fig. S6). Quantitative analysis of NET formation with SYTOX green measurement of relative fluorescence units showed strain-related differences with a higher NET generation after PAO1 exposure compared to D61 (Fig. 5A). This finding was supported qualitatively by SEM imaging where NET formation was more pronounced under PAO1 stimulation (Fig. 5B). Increased NET formation in response to PAO1 might be due to a higher bacterial load in culture supernatants compared to D61 (Supplementary Fig. S7). No age-related differences in NET formation were found though, neither quantitatively, nor qualitatively.

**Fig. 6:**
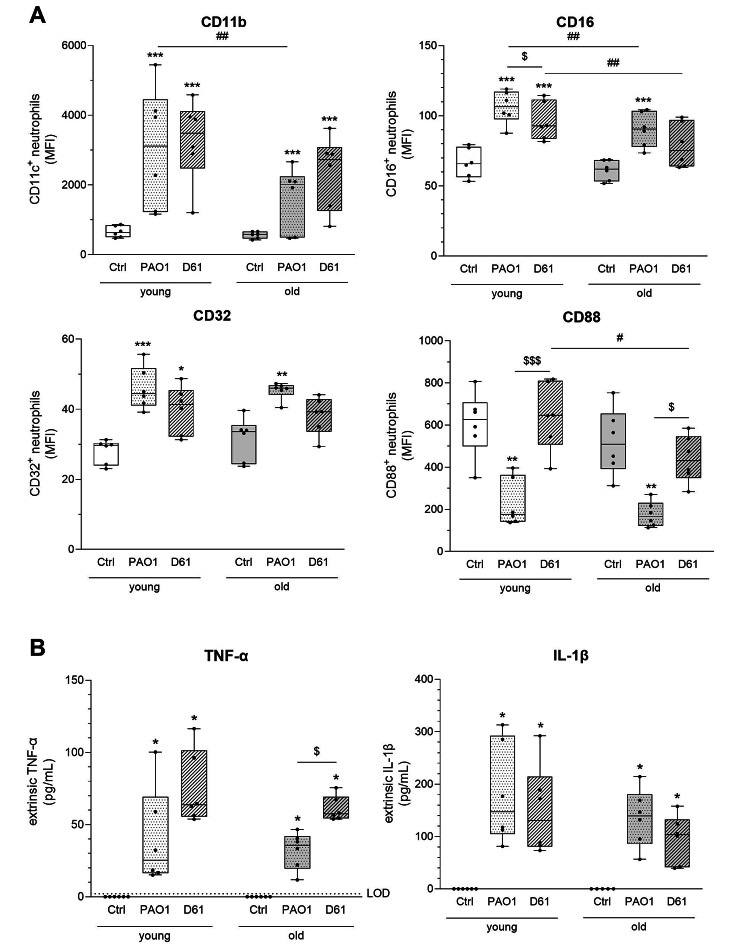
**Neutrophil surface markers are age-dependently regulated upon** ***P. aeruginosa*** **exposure**. Neutrophils of young and old mice were left unstimulated (Ctrl) or stimulated with bacteria (PAO1 or D61) at MOI of 5 for 4 h. **A** Receptor expression of CD11b, CD16, CD32, and CD88 was analyzed using flow cytometry and is depicted as mean fluorescence intensity (MFI). **B** Secretion of TNF-α and IL-1β was analyzed in culture supernatants. n = 6 mice per age group. * *p* < 0.05, ** *p* < 0.01, *** *p* < 0.001, compared with respective control within one age group. # *p* < 0.05, ## *p* < 0.01, between the two age groups. $ *p* < 0.05, $$$ *p* < 0.001, between two bacterial strains within one age group. All other conditions are not significant

In contrast to NET formation, a significant age-dependent difference in surface molecule expression by neutrophils was observable upon *P. aeruginosa* stimulation (Fig. 6A). This was assessed by the expression of neutrophil surface activation markers such as CD11b, CD16, and CD88. In neutrophils isolated from bone marrow of old mice, there was a reduced expression of CD11b and CD16 after PAO1 and a lower CD16 and CD88 expression after D61 exposure compared with neutrophils isolated from young animals. Furthermore, strain-dependent differences were observed with an increased response for CD11b, CD16, and CD32 and a decreased response for CD88 with PAO1 compared with D61 exposure.

Relatively low cytokine expression levels were detected after 4 h of bacterial exposure – of 13 measured cytokines only an increase in early cytokines IL-1β and TNF-α was detected – with strain-specific differences in TNF-α secretion of neutrophils of old mice (Fig. 6B). Nevertheless, the results provide evidence for age- and strain-specific changes in neutrophil responses towards *P. aeruginosa* infection such as neutrophil recruitment and activation which are summarized in Supplementary Table S4.

### Inflammatory response in co-culture experiments

To investigate the impact of neutrophils and PCLS on age-dependent inflammatory responses of uninfected and *P. aeruginosa*-infected lung slices, PCLS from young and old mice were co-cultured with neutrophils isolated from the same mice in a 2 × 2 design and infected with PAO1 for 4 h. Analysis of cytokines indicated a global PCLS age-effect and PAO1 infection-effect for CXCL1, IL-6, and IL-1β, while for TNF-α only a global infection-effect was observed (Supplementary Fig. S8). For IL-1β, a PCLS age-effect was additionally detected within PAO1 infection, indicating that an increased amount of IL-1β is produced by infected PCLS from old compared with young mice after 4 h. However, in the chosen setting, no neutrophil effects were observed in the co-culture system.

## Discussion

The concept of increasing inflammation during aging, referred to as ‘inflamm-aging’, has long been established in the field of aging. Still, the impact of further challenges such as bacterial infections upon inflamm-aging are poorly described. Here, we show that early events of inflamm-aging defined as altered, dysregulated inflammatory responses with aging can be mimicked ex vivo using PCLS and that inflammation during aging is amplified upon *P. aeruginosa* infection of lung slices and neutrophil cultures.

### Inflamm-aging in lung slices of old mice

To depict age-related changes of biological processes in gene expression of PCLS, DAVID gene ontology-enrichment analysis was done on gene array data sets of uninfected control PCLS derived from young and old mice. The results revealed that DEGs were highly associated with immunological processes such as inflammatory response, immune system process, and chemotaxis in PCLS from old compared with young mice. This, hence, provides strong evidence of age-related alteration in the immune response and coincides with the described phenomena of inflamm-aging and immunosenescence [reviewed by 38]. Furthermore, DAVID analysis indicated age-dependent changes in adaptive immune response, underlined by a highly increased expression of genes associated with immunoglobulins, including the common joining chain (*Jchain*), and Fc receptors for IgE and IgG. These findings support results of previous studies reporting increased IgG concentrations in serum and bronchoalveolar lavage of elderly humans (13, 37) and could, together with altered antibody specificities in elderly humans [reviewed in 39], contribute to an increased self-reactivity in aging. Furthermore, detailed analysis of genes and proteins frequently related to inflamm-aging such as IL-6, TNF-α, CXCL1, and IL-1β was done. We measured a significantly increased gene expression of *Il1b*, *Il6*, and *Cxcl1* in PCLS of old compared with young mice. Similar observations were also made in previous studies, showing a significant induction of gene expression of *Il1b*, *Tnfa*, and *Ccl8* in lung tissue of 15-month-old compared with 3-month-old mice [40]. At protein level, we found a significant age-dependent increase in IL-17A. IL-17A is mainly produced by natural killer or T helper 17 cells and supports the recruitment of neutrophils to the site of inflammation [reviewed in 41]. The observed discrepancies between transcriptome and protein data emphasize the complexity of regulatory networks from gene expression to protein synthesis. This could include temporal differences in detection sensitivity or additional translational regulations that are not reflected in the gene expression analysis. Notably though, an age-related increase in inflamm-aging-related cytokines IL-1β, IL-6, and CXCL1 in PCLS of old mice was also observed in neutrophil co-culture experiments that were performed with 3 month and > 26 month old mice (Supplementary Fig. S8), supporting our hypothesis that inflamm-aging can be detected and mimicked in ex vivo lung slices.

### Age-related inflammatory response upon *P. aeruginosa* infection of murine lung slices

Similar to uninfected control PCLS, genes of processes associated with the immune system were strongly regulated with aging upon *P. aeruginosa* infection. Of note, age-dependent differences of cytokines and chemokines increased after *P. aeruginosa* infection on protein level. These differences were mostly significant for D61, despite lower bacterial load compared to PCLS infected with PAO1, indicating an increased immunogenicity of the host-adapted strain. Possibly, mutations in the quorum-sensing gene *lasR* found in D61 [27] alters opsonization, therefore affecting immune responses as shown in a recent study [42]. On gene expression level, age-dependent differences only were observed for *Tnf* and *Il1b* of PAO1-infected lung slices and *Il17a* after infection with either of the two bacterial strains. Generally, our results of age-dependently increased cytokine responses towards bacteria support findings of in vivo stimulation of aged mice using LPS of *P. aeruginosa* or *Escherichia coli*, leading to age-dependently increased levels of TNF-α, IL-1β, IL-6, CCL3, CXCL1, and IL-17A in lung homogenates or bronchoalveolar lavage fluid of challenged mice [43–45].

Increased concentrations of CXCL1, IL-17A, and CCL3 observed in our study could contribute to the enhanced neutrophil infiltration seen in elderly mice stimulated with LPS or infected with *P. aeruginosa* compared with young animals [43–46]. Additionally, within the DAVID analysis, we found (neutrophil) chemotaxis among the top regulated processes with age, further underlining the influence of aging on chemotactic potential of immune cells. Increased gene expression of receptors associated with macrophage or neutrophil chemotaxis in PCLS of old compared to young mice could synergize with enhanced chemokine levels to further promote recruitment of cells into the aging lung and add to increased infiltration of neutrophils under inflammatory stimuli. Together, this might represent a compensatory mechanism for reduced neutrophil pathogen clearance seen in aging [22–24].

### Age-dependent neutrophil activation upon *P. aeruginosa* infection

Based on the crucial role of neutrophils during *P. aeruginosa* infection and our findings in lung tissue hinting towards age-related changes in neutrophil function, we further analyzed activation of the granulocytes upon *P. aeruginosa* stimulation in neutrophil cultures. Recent data showed improved pulmonary immune responses of old mice infected with *Klebsiella pneumonia* upon bone marrow transplantation with cells from young mice [47], hinting that function of bone marrow-derived cells, including neutrophils, declines with age and impacts pulmonary infection. Therefore, we considered bone marrow-derived neutrophils as a suitable model to investigate age-dependent NET formation as well as cytokine release and expression of surface activation markers with and without *P. aeruginosa* infection.

NET formation has been shown to play an important role in innate immunity, as NETs immobilize and neutralize pathogens including bacteria [16]. Although it has been reported previously that neutrophils from aged mice have a reduced NET formation in response to the gram-positive bacterium *S. aureus* [48], we did not see age-dependent changes in NETs towards *P. aeruginosa* which might be due to different mechanisms of NET formation towards the gram-negative *P. aeruginosa*. However, we found NET formation of neutrophils in response to the clinical isolate D61 to be lower as compared to PAO1. These findings support previous reports stating that NET release of human neutrophils is lower when using late clinical *P. aeruginosa* isolates compared to early isolates of the same cystic fibrosis patient [49]. Lower NET formation might be accounted for by a reduced bacterial growth of late clinical *P. aeruginosa* isolates as compared to early isolates or PAO1 which was found by us and others [50, 51]. Furthermore, NET formation is reported to be reduced towards *P. aeruginosa* strains with mutations in the master regulator *lasR* [52], such as found for D61 [27], which might further explain differences in NET formation and hints towards immune evasion of the host-adapted strains.

Besides NET formation, we also addressed cytokine release and expression of surface activation markers in neutrophils isolated from young and old mice. In contrast to PCLS, no age-related differences were found in cytokines released by neutrophils and the cytokine response was generally low – only the very early pro-inflammatory cytokines IL-1β and TNFα were detectable 4 h after infection with *P. aeruginosa*. This might be explained by the early time point and the fact that neutrophils primarily respond to TNF-α and IL-1β, but are not described as main producers of these cytokines [49] – in contrast to alveolar macrophages that reside in PCLS [53, 54]. Nevertheless, significant differences were found in surface activation molecules with age and infection. A decline in CD11b (integrin α M, ITGAM) and CD16 (FcγRIII) was present on neutrophils from old compared with young mice upon stimulation with *P. aeruginosa*. CD11b is both a complement receptor (CR3) and a cell adhesion molecule that is required for cell recruitment to the site of inflammation and CD16 is involved in phagocytosis and neutrophil degranulation. These surface molecules are generally upregulated in inflammation and neutrophil activation [55], which we also observed after ex vivo stimulation with *P. aeruginosa*, although to a lesser extend in neutrophils from old mice. In human blood neutrophils, levels of CD16 expression were reported to be decreased in comparison to younger donors [55, 56], and levels of CD11b neutrophil expression were reported to be either unaffected [56] or decreased [55] with aging. Upon LPS challenge of neutrophils from mice, however, similar observations of a reduced CD16 and CD11b activation with increasing age were made [55], confirming the findings of our results. Additionally, a decreased stimulation was found in neutrophils from old compared with young mice in response to exposure with the D61 strain for CD88 (C5AR1; complement component 5a receptor). Together, these results provide evidence that ex vivo activation of neutrophils from old mice was reduced upon bacterial challenge. These findings are in contrast to the observation of increased gene expression levels in the PCLS of old mice upon exposure with *P. aeruginosa*, where *C5ar1*, *Fcer1g*, *Fcgr3, Itgb2*, and *Itgam* were measured to be up-regulated. Both findings are, however, in line with the literature and provide evidence that inflamm-aging in the lung is increased with age, but neutrophil activation without additional stimulation of pulmonary and systemic immune response is reduced. This most likely complements the phenotype of immunosenescence as observed in in vivo studies in old mice with pulmonary infection [46, 57], where an increased inflammation and neutrophil recruitment with lowered bacterial killing add to the pathology of lung injury. Nevertheless, it has to be considered that the PCLS model can only mimic very early inflammatory responses of infection since recruitment of inflammatory cells including neutrophils, monocytes, and lymphocytes is lacking. Our pilot experiment combining infected PCLS with neutrophils in a co-culture model did not indicate any influence of neutrophils on cytokine production. This could, however, also be due to experimental settings that do not allow cell recruitment over time, as static co-culture models can only poorly mimic complex spatial and temporal interactions. Future studies combining PCLS and isolated neutrophils in dynamic co-culture models, such as provided in organ-on-chip systems, could therefore help to unravel the impact of neutrophil recruitment and immunological mechanisms in age-dependent differences of *P. aeruginosa*-induced pneumonia.

## Conclusions

**Fig. 7:**
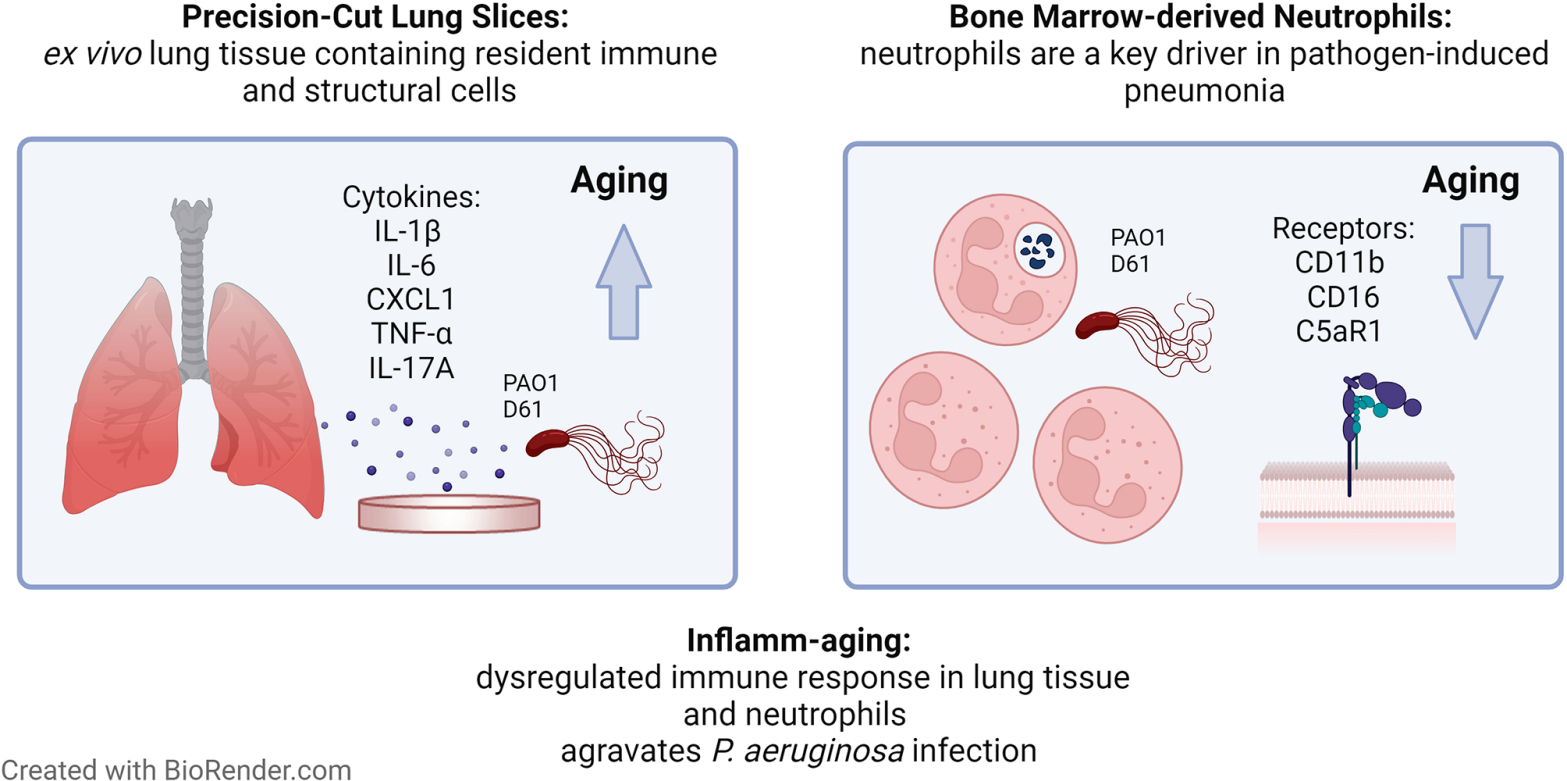
**Inflamm-aging modulates the immune response towards** ***P. aeruginosa*** **infection in PCLS and neutrophils**. In summary, cytokine responses were found to be increased in PCLS from old compared to young mice upon infection with *P. aeruginosa* PAO1 or a clinical isolate D61. On isolated neutrophils, receptor expression upon bacterial infection was age-dependently reduced. Both findings highlight the changes of the immune system with aging that are crucial for pulmonary infections

In summary, our results provide new evidence that very early events of pulmonary inflamm-aging can be mimicked ex vivo in tissue slices of distal lungs and that aging promotes pulmonary inflammation upon *P. aeruginosa* infection. The results presented here provide mechanistic insights into first host responses to *P. aeruginosa* infection. These were particularly characterized by an increased production of pro-inflammatory cyto- and chemokines in lung tissue with advancing age, similarly as in murine in vivo models or human samples, indicating that the model is well suited for ex vivo investigations of pulmonary infections in aging. Furthermore, ex vivo neutrophil activation in *P. aeruginosa* infection was also impaired with aging as shown by a decline in surface receptors. These findings (summarized in Fig. 7) complement the phenotype of immunosenescence and inflamm-aging that promotes enhanced tissue damage and severity of *P. aeruginosa* pneumonias in the elderly. In turn, rebalancing immune responses in aging patients might reduce the incidence of severe pneumonias and associated mortality rates.

### Electronic supplementary material

Below is the link to the electronic supplementary material.

Supplementary Material 1

Supplementary Material 2

## Data Availability

The datasets supporting the conclusions of this article are included within the article and its supplementary information files. Raw transcriptome data were deposited at GEO database (GSE208375).

## List of Abbreviations

BCA: Bicinchoninic acid
C5AR1: Complement component 5a receptor
CR: Complement receptor
DAVID: Database for Annotation, Visualization and Integrated Discovery
DEG: Differentially expressed gene
IL: Interleukin
ITGAM: Integrin α M
MOI: Multiplicity of infection
NET: Neutrophil extracellular trap
PCLS: Precision-cut lung slices
p.i.: Post infection
SEM: Scanning electron microscopy
TNF-α: Tumor necrosis factor-α
