# Supplementary material for: Age-dependent inflammatory response is altered in an ex vivo model of bacterial pneumonia
Source: Respir Res. 2024 Jan 4;25:15. doi: 10.1186/s12931-023-02609-w (PMC10765774; doi:10.1186/s12931-023-02609-w)
Supplement: Supplementary file 2 — Supplementary Material 2 [file 12931_2023_2609_MOESM2_ESM.docx]

**Age-dependent inflammatory response is altered in an *ex vivo* model of bacterial pneumonia**

- Online Supplements -

# Supplementary methods

## Bacterial strains

The strain D61 was isolated after approximately 15 years of chronic colonization in the lung of a female cystic fibrosis patient with a mild clinical course, where a chronic colonization with *P. aeruginosa* occurred at age 19. D61 expresses functional pili and a flagellum, thereby mediating motility (Klockgether et al., 2018).

Lysogeny-broth (LB) medium (Carl Roth) was inoculated with *P. aeruginosa* glycerol stocks and incubated overnight at 37 °C, 150 rpm. Overnight cultures of bacteria were centrifuged at 3,000 g for 10 min and re-suspended in DMEM/F-12 (Gibco by Life Technologies). Bacterial suspensions were adjusted to the required inoculum using OD_600_.

## Preparation and infection of PCLS

According to previous literature (Held et al., 1999; Henjakovic et al., 2008), murine lungs were filled with agarose. Separate lobes were cut into slices of 320 µm thickness in ice-cold Earle’s Balanced Salts Solution (Sigma-Aldrich) using an automatic oscillating microtome (7000smz‑2, Campden Instruments, Loughborough, England). Tissue slices were transferred into ice-cold DMEM/F-12 and washed for 30 min in DMEM/F‑12 three times at 37 °C, 5% CO_2_ to remove cell debris.

Bacteria were added to two PCLS/well. One hour post infection (p.i.), the inoculum was discarded and PCLS were washed once with warm Dulbecco's phosphate-buffered saline (DPBS, Lonza, Basel) and transferred into new plates containing DMEM/F-12. Tissue cultures were post-incubated for 7 h at normal cell culture conditions. Serial dilutions of supernatants were plated on LB-agar plates (Becton Dickinson Labware) and CFUs were counted. Tissue was washed and lysed in 1% Triton X-100 in DPBS (Sigma-Aldrich) for 30 min at 4 °C, followed by vigorous mixing to detach bacteria from the tissue matrix and CFU of lysates was determined. Lysates furthermore were used to determine total protein content using the Pierce^Tm^ BCA Protein Assay Kit (Thermo Fisher Scientific) (measured in duplicates). To determine viability, PCLS were stained with Calcein-acetoxymethylester (AM) (C3100MP, Thermo Fisher) as previously described (Neuhaus et al., 2018) with some modifications: After staining, tissue was lysed with 1 % Triton-X in DPBS for 30 min at 150 rpm to release the fluorescent dye. Fluorescence of lysates was measured in duplicates in black 96-well plates using i-control (Tecan Group, version 1.10) and the Infinite^®^ 200 Pro microplate reader (Tecan Group) (excitation: 485 nm, emission: 535 nm).

## Dissociation of PCLS

To prepare single-cell suspensions for flow cytometrical analysis, 2 PCLS were finely minced in 24-well plates using micro scissors and 500 µl of dissociation medium (DMEM-F12 + 1 % pen/strep + 1 mg/ml Collagenase D + 0.055 mg/ml DNase I) were added. PCLS were dissociated for 60 min at 37 °C while shaken (150 rpm). The plate was placed on ice to stop the reaction and the suspension was extensively pipetted up and down using a cut pipette tip to further mechanically dissociate the tissue. The cell suspension was filtered through a 100 µm cell strainer and the strainer was rinsed with 3 ml DMEM-F12. After centrifugation, the supernatant was aspirated and the cell pellet was further processed for flow cytometrical staining.

## *P. aeruginosa* growth kinetics

For growth kinetics of PAO1 and D61, 1x10^5^ CFU were incubated in 500 µl DMEM-F12 for 24 h at 37 °C in the Tecan reader. Optical density was measured every 30 min and the plates were shaken briefly before each measurement.

## RNA isolation and transcriptomics

RNA isolation protocol was based on a specific homogenization and phenol extraction procedure coupled with a MagMax™ magnetic beads (ThermoFisher Scientifc) cleaning procedure. RNA integrity number (RIN) was quantified (Agilent 2100 Bioanalyzer®; Agilent Technologies) revealing a good quality with high RIN values between 7.6 and 9.5 for all samples.

A total RNA of 100 ng was used as a starting material for target preparation. Arrays were subsequently washed, stained, and scanned using the Affymetrix GeneChip™ Command Console Software (ThermoFisher Scientific) with .cel files as data output. Raw data were deposited at GEO database (GSE208375). Subsequent quality control, normalization, and visualization of microarray data were undertaken using metrics and methods contained in Transcriptome Analysis Console Software (TAC 4.0, Thermo Fisher Scientific). The full list of DEGs can be found in the supplementary information (SI 1).

## Quantification of NETs

To quantify NET formation, 100,000 neutrophils were incubated with bacteria at MOI 5 in DMEM/F-12 in black 96-well microtiter plates. A PMA and ionomycin cell activation cocktail (423301, BioLegend) served as a positive control. DNase I (100 U/ml) was added to PMA/ionomycin-stimulated neutrophils to degrade NETs (Sigma-Aldrich). Extracellular DNA was stained by 1.5 µM SYTOX green (Thermo Fisher). Fluorescence was measured in duplicates at 37 °C after 4 h using the Tecan reader and (excitation: 485 nm, emission: 535 nm). Background SYTOX-signal by medium (without neutrophils or bacteria) was subtracted from all other measurements and the ratio of the sample’s SYTOX-signal to unstimulated control neutrophils of the same mouse was calculated.

## Scanning electron microscopy of NETs

To visualize NETs using Scanning electron microscopy, 300,000 neutrophils were allowed to attach to cover slips for 1 h at 37 °C. Bacteria were added at a MOI of 10 and the co-cultivation was performed under normal cell culture conditions. After 4 h, cells were fixed overnight at 4 °C in 1.5% glutaraldehyde and 1.5% paraformaldehyde in 0.15 M HEPES buffer. The samples were further processed for SEM imaging by dehydration in acetone, critical point drying and gold sputtering (Schröder et al., 2020).

## Immunofluorescence of NETs

To visualize NET components, 300,000 neutrophils were allowed to attach per laminin-coated cover slip (18 mm, Neuvitro Corp., Vancouver, USA) for 1 h at 37 °C. Bacteria were added at a MOI of 5 and the co-cultivation was performed over 4 h under normal cell culture conditions. PMA/Ionomycin was added as a positive control. After 4 h, cells were fixed with paraformaldehyde (PFA, Sigma-Aldrich) at 4 °C overnight. On the next day, cover slips were washed in DPBS following blocking at room temperature with 4% normal donkey serum (Jackson ImmunoResearch), diluted in DPBS. Cells were stained with a goat anti-mouse myeloperoxidase antibody (AF3667, R&D) diluted in donkey serum for 1 h at room temperature. After washing, the secondary antibody was added for 45 min in the dark (Cy3 donkey anti-goat antibody). Cover slips were washed, stained with DAPI and mounted onto glass slides using ProLong Antifade (P36930, Thermo Fisher). NETs were visualized using an Axio Scan Z.1 microscopic slide scanner (Carl Zeiss Microscopy GmbH).

## Flow cytometry

After bacterial exposure, neutrophils were centrifuged. Neutrophils and single-cells suspensions from PCLS were blocked with PBS, 5% FCS and CD16/32 antibody (#101301, BioLegend) for 15 min on ice, followed by incubation with antibody mixture, including single-staining controls for compensation (for neutrophils CD32-FITC 1:200, CD88-PE 1:200, CD11c-PerPC 1:200, CD16-APC 1:100, CD62L-PECy7 1:200; all from BioLegend; for PCLS eFluor™ 506 fixable viability dye 1:500 from Thermo Fisher Scientific, EpCAM-FITC 1:200, CD31-APC 1:100, CD45-PE-Cy7 1:100; all from BioLegend) in PBS containing 2% FCS, on ice in the dark. After 30 min, neutrophils were fixated with 4% PFA, followed by washing with PBS and storing cells overnight in the dark at 4°C. Single-cell suspensions of PCLS were measured immediately after adding CountBright™ counting beads (Thermo Fisher Scientific) to determined cell numbers per 2 PCLS. Flow cytometric analysis was done with a FACS Canto II flow cytometer (BD Biosciences) for neutrophils and with a CytoFLEX S (Beckman Coulter) for PCLS. Subsequent analysis was done with the FlowJo software (FlowJo version10.7.2).

# References

Held HD, Martin C, Uhlig S. Characterization of airway and vascular responses in murine lungs. *Br J Pharmacol* 1999;126:1191–9.

Henjakovic M, Sewald K, Switalla S, Kaiser D, Müller M, Veres TZ, Martin C, Uhlig S, Krug N, Braun A. Ex vivo testing of immune responses in precision-cut lung slices. *Toxicol Appl Pharmacol* 2008;231:68–76.

Klockgether J, Cramer N, Fischer S, Wiehlmann L, Tümmler B. Long-Term Microevolution of Pseudomonas aeruginosa Differs between Mildly and Severely Affected Cystic Fibrosis Lungs. *Am J Respir Cell Mol Biol* 2018;59:246–56.

Neuhaus, V., Danov, O., Konzok, S., Obernolte, H., Dehmel, S., Braubach, P., Jonigk, D., Fieguth, H. G., Zardo, P., Warnecke, G., Martin, C., Braun, A., Sewald, K. Assessment of the Cytotoxic and Immunomodulatory Effects of Substances in Human Precision-cut Lung Slices. J. Vis. Exp (135), e57042, doi:10.3791/57042 (2018).

Schröder M-L, Angrisani N, Fadeeva E, Hegermann J, Reifenrath J. Laser-structured spike surface shows great bone integrative properties despite infection in vivo. *Mater Sci Eng C Mater Biol Appl* 2020;109:110573.

# Supplementary Tables

**Table S1**: Top 5 down- and upregulated genes of uninfected control PCLS of old compared to young mice (*p* < 0.05). Predicted genes were excluded.

| **Gene** | **Description** | **fold change** |
| --- | --- | --- |
| Jchain | immunoglobulin joining chain | 26.56 |
| Fcgr2b | Fc receptor, IgG, low affinity IIb | 6.53 |
| Itgam | integrin alpha M | 5.98 |
| Fcer1g | Fc receptor, IgE, high affinity I, gamma polypeptide | 5.11 |
| Irf7 | interferon regulatory factor 7 | 4.81 |
| Sly | Sycp3 like Y-linked | -2.76 |
| Sly | Sycp3 like Y-linked | -2.87 |
| Sln | sarcolipin | -3.19 |
| Ipw; Snord116 | imprinted gene in the Prader-Willi syndrome region; small nucleolar RNA, C/D box 116 cluster | -4.22 |
| Gm20871 | Sycp3 like Y-linked pseudogene | -4.62 |

**Table S2**: Top 5 down- and upregulated genes of PCLS infected with PAO1 of old compared to young mice (*p* < 0.05).

| **Gene** | **Description** | **fold change** |
| --- | --- | --- |
| Jchain | immunoglobulin joining chain | 128.47 |
| Fcgr2b | Fc receptor, IgG, low affinity IIb | 5.69 |
| Gbp2b; Gbp5 | guanylate binding protein 2b; guanylate binding protein 5 | 4.67 |
| Hcar2 | hydroxycarboxylic acid receptor 2 | 4.25 |
| Gzmk | granzyme K | 4.17 |
| Dpt | dermatopontin | -2.63 |
| Nckap5 | NCK-associated protein 5 | -2.64 |
| Myh7 | myosin, heavy polypeptide 7, cardiac muscle, beta | -2.75 |
| Kdr | kinase insert domain protein receptor | -2.89 |
| Fmo1 | flavin containing monooxygenase 1 | -3.21 |

**Table S3**: Top 5 down- and upregulated genes of PCLS infected with D61 of old compared to young mice (*p* < 0.05).

| **Gene** | **Description** | **fold change** |
| --- | --- | --- |
| Jchain | immunoglobulin joining chain | 99.62 |
| Cxcl9 | chemokine (C-X-C motif) ligand 9 | 5.72 |
| Gzmk | granzyme K | 5.01 |
| Fcgr2b | Fc receptor, IgG, low affinity IIb | 4.90 |
| Pla2g7 | phospholipase A2, group VII (platelet-activating factor acetylhydrolase, plasma) | 4.74 |
| Ereg | epiregulin | -2.58 |
| Lamc1 | laminin, gamma 1 | -2.69 |
| Dpt | dermatopontin | -2.70 |
| Nrcam | neuronal cell adhesion molecule | -2.75 |
| Fmo1 | flavin containing monooxygenase 1 | -2.79 |

**Table S4:** Genes and proteins found to be significantly regulated with age in uninfected (Ctrl) or PAO1- or D61-infected PCLS, neutrophil cultures, or PCLS-neutrophil co-cultures (old vs. young *p* < 0.05). For differentially regulated genes, genes encoding for receptors associated with neutrophil chemotaxis and chosen cytokines are depicted.

|  | **PCLS**  8 h | | | **Neutrophils**  4 h | **Co-cultures**  4 h |
| --- | --- | --- | --- | --- | --- |
| Age effect in treatment groups | Gene expression | | Protein expression | Protein expression | Protein expression |
|  | Neutrophil receptors | Cytokines | Cytokines | Receptors | Cytokines |
| Ctrl | *Fcgr3, Itgam, C5ar1, Fcer1g, Itgb2* ↑ | *Il1b, IL6,*  *Cxcl1* ↑ | IL-17A ↑ |  |  |
| PAO1 | *Fcgr3, Itgam, C5ar1, Fcer1g, Itgb2, Trem1* ↑ | *Tnf, Il1b,*  *Il17a* ↑ | IL-1β, IL-17A ↑ | CD11b, CD16 ↓ |  |
| D61 | *Fcgr3, Itgam, C5ar1, Fcer1g, Itgb2, Trem1* ↑ | *Il17a* ↑ | TNF-α, IL-1β, IL-6, CCL3, CCL20, CXCL1,  IL‑17A ↑ | CD16, CD88 ↓ |  |
| Global age-effect |  |  |  |  | IL-1β, IL-6, CXCL1 ↑ |

# Supplementary Figures


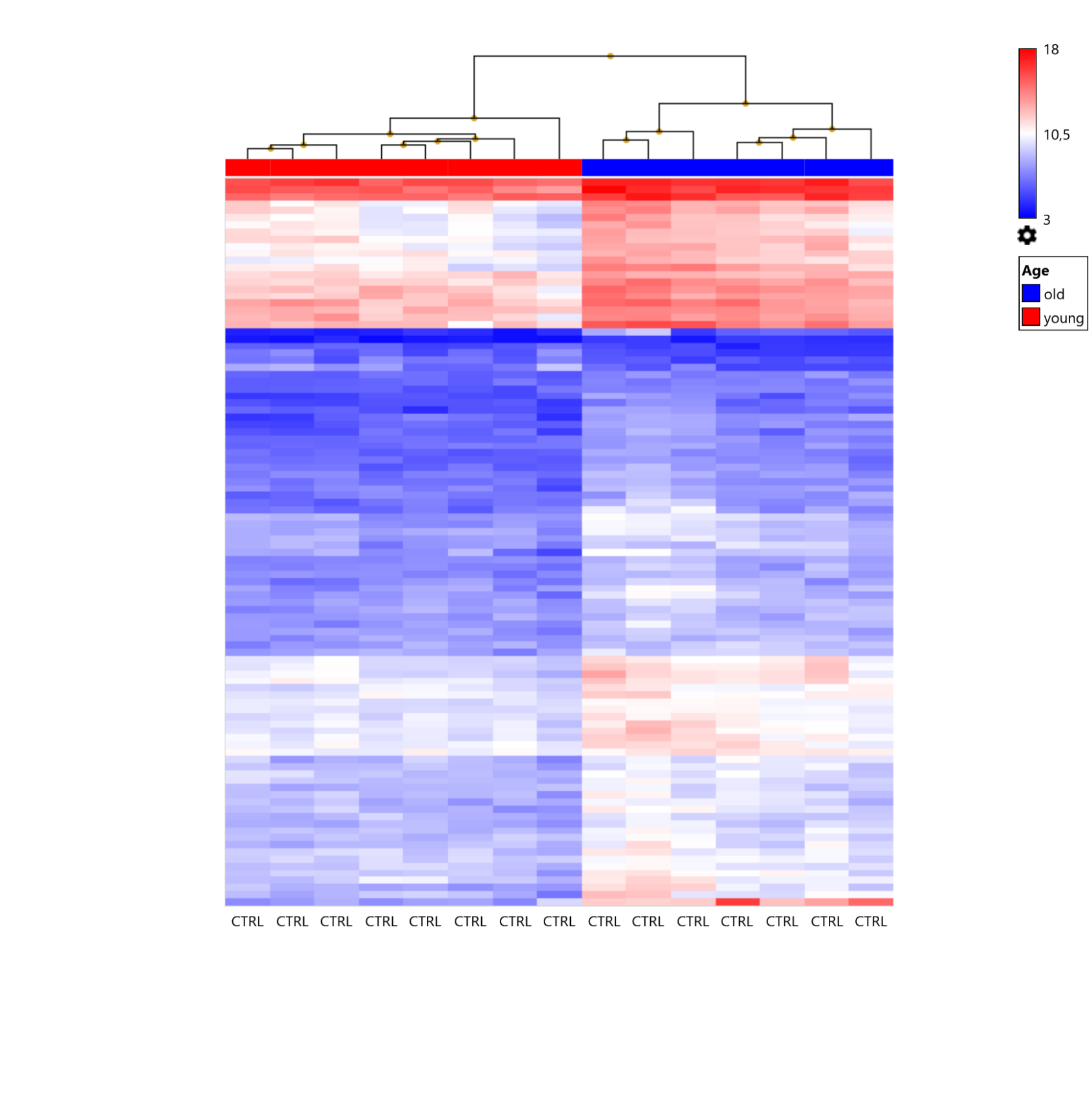


**Figure S1. Gene expression of *ex vivo* lung tissue differs between young and old mice.** PCLS of young and old mice were cultured for 8 h (2 PCLS/well) and whole genome analysis was performed. Overview of gene expression analysis depicted as unsupervised hierarchical clustering (≥ 2-fold change, *p* < 0.05, FDR *p* < 0.05).


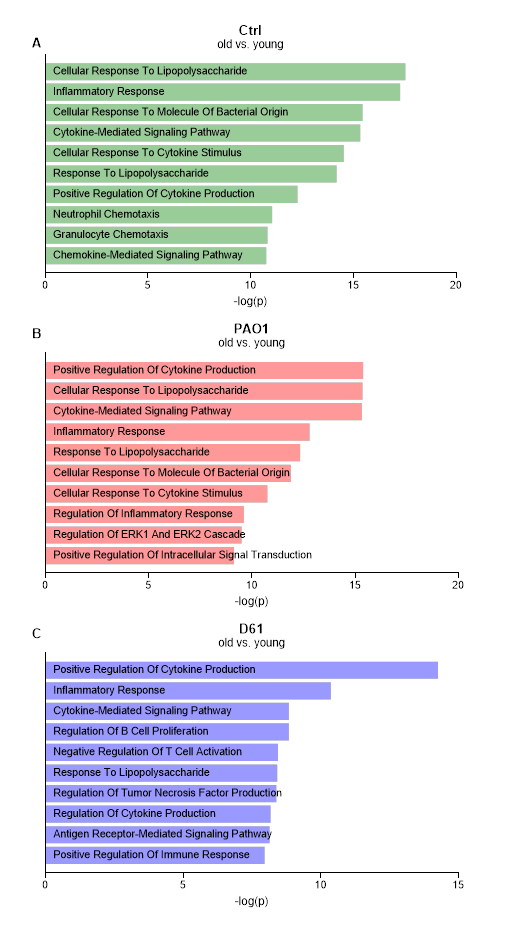


**Figure S2. Biological processes specifically affected by age include immune-related processes in uninfected and *P. aeruginosa*-infected PCLS analyzed with Enrichr**. PCLS of young and old mice were infected with 1x10^5^ CFU/well PAO1 or D61 or cultured without bacteria (Ctrl). After 8 h, whole genome analysis was performed. Differentially regulated genes (≥ 2‑fold change, *p* < 0.05) were analyzed in Enrichr for enrichment analysis of biological process ontology. The top 10 biological processes are displayed for Ctrl (**A**), PAO1-infected (**B**), and D61-infected PCLS (**C**), ranked based on their *p*-value.


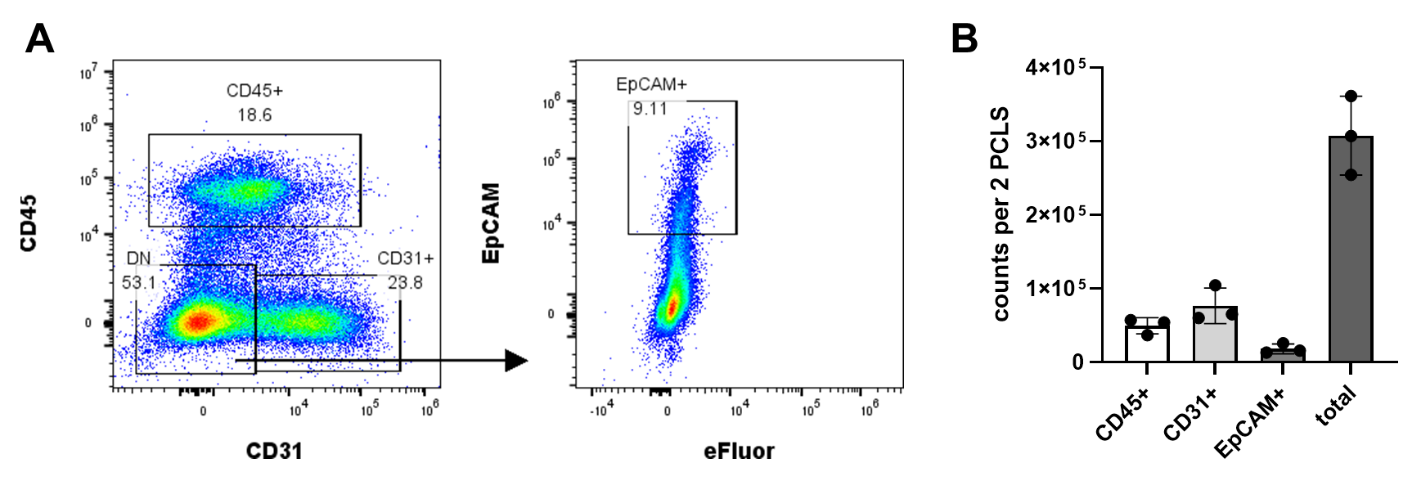


**Figure S3.** **Counts of cell subsets in murine PCLS.** PCLS were prepared from mice and dissociated for flow cytometrical analysis of single-cell suspensions. **A** Representative gating strategy for single, viable cells. **B** Counts of respective cells determined in 2 PCLS. Total: all single, viable cells. n = 1 mouse, technical triplicates.

**Figure S4. Protein content of *P. aeruginosa*-infected and uninfected PCLS.** PCLS of young and old mice were infected with PAO1 or D61 for 8 h or left unstimulated (Ctrl). Tissue lysates were collected and used for determination of total protein content. n = 8 mice per age group.


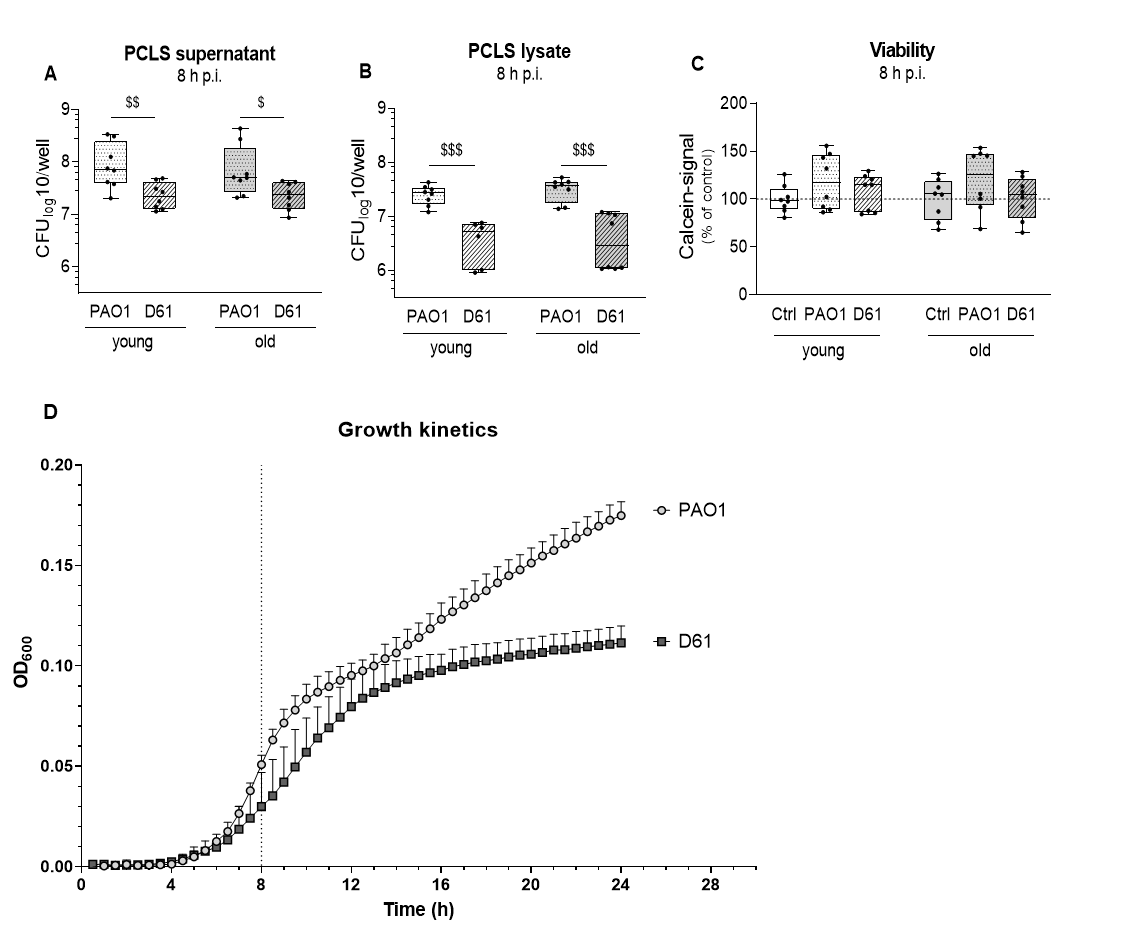


**Figure S5.** **Lower growth rates of D61 compared to PAO1. A** and **B** PCLS of young and old mice were infected with 1x10^5^ CFU/well PAO1 or D61 for 8 h. Tissue supernatant and lysates were collected and used for determination of viable bacteria using colony-forming units (CFU) assay. **C** Calcein-AM staining was performed to determine viability of the tissue. Calcein-stained PCLS were lysed and relative fluorescence units of lysates were determined in duplicates. Mean of duplicates was normalized to respective uninfected control PCLS (Ctrl). n = 8 mice per age group, technical duplicates (Ctrl) or triplicates (PAO1 and D61), 2 PCLS/well. $ *p* < 0.05, $$ *p* < 0.01, $$$ *p* < 0.001, between two bacterial strains within one age group. Differences between the two age groups were not significant. Box plots display the median with the 25^th^ and 75^th^ percentile. Whiskers mark the lowest and highest value. **D** For growth kinetics**,** 1x10^5^ CFU/well PAO1 or D61 were cultured for 24 h in DMEM/F-12 and optical density (OD_600_) was measured every 30 min. n = 2 independent experiments, measured in quadruplicates. Dashed line marks 8 h time point used for infection of PCLS.


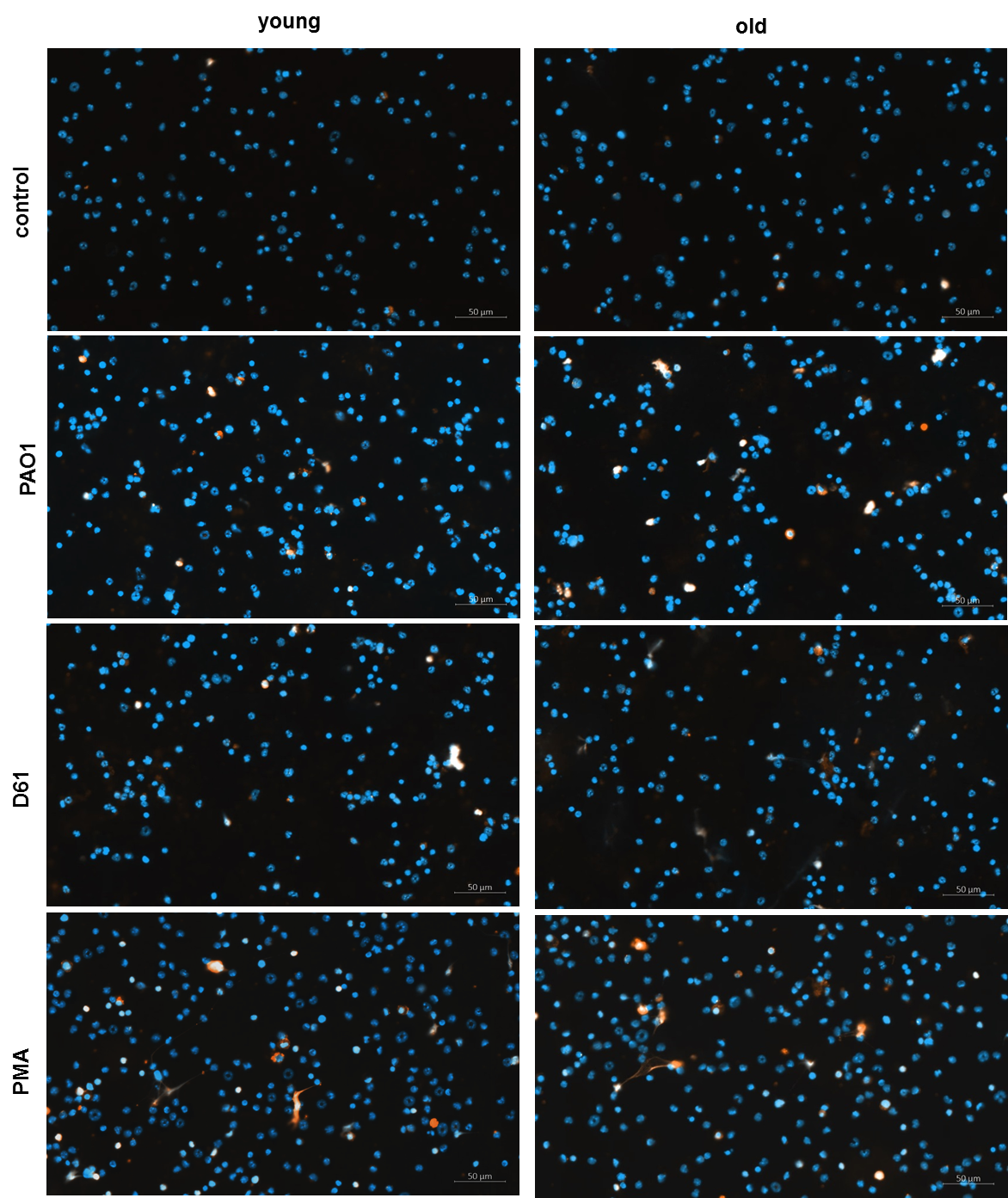


**Figure S6. Fluorescent microscopic images of NET formation.** DAPI signal (blue) shows nuclei and myeloperoxidase signal (orange) extracellular NET formation. Increasing NET formation was found with bacterial exposure and PMA/ionomycin exposure (positive control) in neutrophils isolated from young and old mice.

**Figure S7. Bacterial load of clinical *P. aeruginosa* isolate is lower compared to PAO1 when cultured with neutrophils of mice from different ages.** Neutrophils of young and old mice were cultured with bacteria (PAO1 or D61) at MOI of 5 for 4 h. Cell supernatants were collected and used for determination of viable bacteria using colony-forming units (CFU) assay. n = 6 mice per age group, technical duplicates. $$$ *p* < 0.001, between bacterial strains within one age group. No differences between the two age groups. Box plots display the median with the 25^th^ and 75^th^ percentile. Whiskers mark the lowest and highest value.


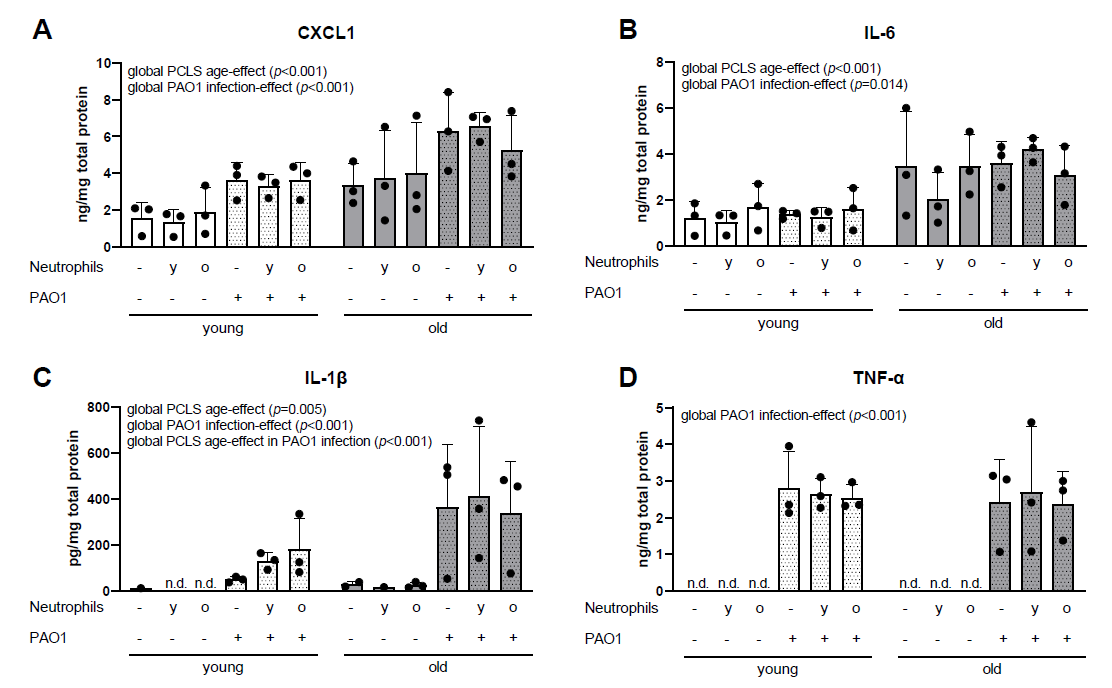


**Figure S8. Cytokine production in co-cultures of PCLS and neutrophils from young and old mice is not altered after 4 h.** PCLS from young and old mice were co-cultured with 100,000 neutrophils from young (y) and old (o) mice in a 2 x 2 design and infected with 1x10^5^ CFU PAO1. After 4 h, supernatants and tissue lysates were harvested for analysis of CXCL1 (**A**), IL-6 (**B**), IL-1β (**C**), and TNF-α (**D**). Total cytokine content (extrinsic and intrinsic) was normalized to the total protein content. n = 3 mice per age group, analyzed by 3-way ANOVA to test for age-effects in PCLS, age-effects in neutrophils, and PAO1-infection effects (*p*<0.05).
